# Definitive treatment in squamous cell carcinoma of head and neck: A retrospective analysis of chemoradiotherapy in a university hospital setting

**DOI:** 10.1016/j.bjorl.2025.101576

**Published:** 2025-04-02

**Authors:** Hádila Silva Veras Sousa, Vivian Naomi Horita, Davi Magalhães Leite Novaes, Matheus Yung Perin, Daniel Naves Araújo Teixeira, Joyce Gruenwaldt, Eduardo Baldon Pereira, Carlos Takahiro Chone, Gustavo Jacob Lourenço, Ligia Traldi Macedo, Carmen Silvia Passos Lima

**Affiliations:** a Universidade Estadual de Campinas (UNICAMP), Faculdade de Ciências Médicas (FCM), Departamento de Anestesiologia, Oncologia e Radiologia, Serviço de Oncologia Clínica, Campinas, SP, Brazil; b Universidade Estadual de Campinas (UNICAMP), Faculdade de Ciências Médicas (FCM), Departamento de Oftalmologia e Otorrinolaringologia, Campinas, SP, Brazil; c Universidade Estadual de Campinas (UNICAMP), Faculdade de Ciências Médicas (FCM), Departamento de Anestesiologia, Oncologia e Radiologia, Serviço de Radioterapia, Campinas, SP, Brazil; d Universidade Estadual de Campinas (UNICAMP), Faculdade de Ciências Médicas (FCM), Laboratório de Genética do Câncer, Campinas, SP, Brazil

**Keywords:** Head and neck squamous cell carcinoma, Definitive therapy, Outcome

## Abstract

**Objective:**

To evaluate toxicities, tumor control, Event-Free Survival (EFS) and Overall Survival (OS) of patients with locoregionally advanced Head and Neck Squamous Cell Carcinoma (HNSCC) treated with definitive platinum-based Chemoradiation (CTRT).

**Methods:**

A total of 233 patients underwent treatment with RT plus weekly or every three weeks Cisplatin (CDDP) or Carboplatin (Carbo). Toxicity and response to treatment were classified using conventional criteria. Kaplan-Meier, log-rank test, and Cox regression (univariate and multivariate) were used to assess patient survival.

**Results:**

Half of patients presented toxicities grade 3 or 4, with nausea/vomiting and nephrotoxicity being more common in RT and CDDP group and anemia and neutropenia in RT and Carbo group. Complete or partial response was observed in 75% of patients, and the distinct protocols did not alter the treatment response. Two-year EFS and OS probabilities were 43.3% and 66.0%, respectively. Active smoking, an ECOG score of 2 or higher, stage IV tumor, and treatment with RT and Carbo were independent prognostic factors for poorer outcomes. Patients of these groups had approximately double chance of relapse and progression to death compared to others.

**Conclusion:**

Our data indicate definitive treatment with RT and CDDP as the best treatment for locoregionally advanced HNSCC treated in Brazilian public hospitals. However, prospective randomized studies are required to establish the ideal treatment for those patients.

**Level of evidence:**

3.

## Highlights

• RT plus CP or Carbo has been used as definitive treatment for advanced SCCHN.
• Efficacy of protocols in SCCHN patients of developing countries are unknown.
• We analyzed outcomes of advanced SCCHN patients treated with both protocols.
• Patients treated with RT plus Carbo had double chance of relapse and death.
• CP was the best agent for advanced SCCHN in economically limited settings.

## Introduction

Head and Neck Squamous Cell Carcinoma (HNSCC) ranks as the seventh most prevalent cancer globally, with 878,348 new cases and 364,339 annual deaths.^1^ In the Brazilian context, the National Cancer Institute (INCA) estimates 23,000 new cases per year and 11,000 deaths for the period 2023‒2025, placing it as the eighth most incident tumor.^2^ The incidence of HNSCC is notably higher among men, smokers, alcohol users,^3^, ^4^, ^5^, ^6^ and individuals with oral Human Papillomavirus (HPV) infection.^6^, ^7^, ^8^, ^9^ The Overall Survival (OS) for HNSCC patients is approximately 40%‒50% over five years.^10^, ^11^

The management of HNSCC requires a diversified approach, taking into consideration the tumor stage and primary site.^12^, ^13^, ^14^ Surgical Resection (SR) and Radiotherapy (RT) are therapeutic options for patients with localized tumors,^15^ while cases of metastatic tumors often necessitate Chemotherapy (CT).^16^ However, over 60% of HNSCC cases present as locoregionally advanced tumors,^11^, ^17^ signaling the urgent need for multimodal therapeutic strategies.^13^, ^18^ In this scenario, patients with resectable tumors may undergo SR, with or without adjuvant RT or Chemoradiotherapy (CTRT). Patients with unresectable tumors or those refusing surgery due to potential sequelae are typically treated with platinum-based agents such as Cisplatin (CDDP) or Carboplatin (Carbo) in conjunction with RT.^19^, ^20^

Approximately 40% of patients and 30% of patients treated with platinum-based CTRT as definitive treatment obtain Complete Response (CR) or Partial Response (PR) and remain alive at five years of follow up in countries in the northern hemisphere,^16^, ^19^, ^21^ but data on the outcomes of these patients in Brazilian public hospitals are scarce. This study assessed patients diagnosed with locoregionally advanced HNSCC, undergoing platinum-based CTRT as definitive protocol in a public hospital in the southeast region of Brazil. The objectives of the study included analyzing toxicities, Response Rates (RR), and OS, as well as investigating the influence of clinicopathological factors on survival.

## Methods

### Population and selection

This retrospective cohort study focused on patients diagnosed with locoregionally advanced HNSCC treated with definitive CTRT at the Oncology Service of the Oncology Service of the General Hospital of the State University of Campinas (UNICAMP) between January 1, 2015, and December 31, 2019. Inclusion criteria encompassed patients aged 18 or older, with an Eastern Cooperative Oncology Group (ECOG) Performance Status (PS) of 2 or lower at the time of diagnosis. Exclusion criteria included nasopharyngeal tumors, initiation of oncologic treatment before the data collection period, distant metastases prior to treatment, pregnancy, history of another malignancy, severe comorbidities, lack of information in medical records, and refusal to participate in the study. Clinical information, such as age at diagnosis, gender, ECOG Performance Status (PS), smoking history, alcohol consumption, and comorbidities, as well as tumor information, including location, histological grade and stage were obtained from the medical records of all enrolled patients. Patients were categorized based on smoking^22^ and drinking^3^ history into current, former, or never users according to established classification criteria. Diagnosis of HNSCC and histological grading of the tumor were based on World Health Organization criteria^23^ and the criteria of American Joint Committee on Cancer (AJCC) were used to define tumor stage.^14^

### Treatment aspects

All 233 participants in this study underwent platinum-based definitive CTRT as the primary treatment. This strategy was motivated by factors such as the unresectability of the tumor, patient refusal due to potential anatomical and functional losses associated with surgery, or adherence to an organ preservation protocol. The treatment consisted of 35 sessions of RT (2 Gy per session),^24^ administered concurrently with CDDP or Carbo in different regimens. For the CDDP every three weeks, the intravenous dose was 80‒100 mg/m^2^ on days 1, 22, and 43, with 100 mg/m^2^ for ECOG 0 or 1 patients without comorbidities, and 80 mg/m^2^ for others, with dose adjustments or CT suspension in the case of grade 3 or 4 adverse events. In the weekly CDDP group, the dose was 40 mg/m^2^ on days 1, 8, 15, 22, 29, and 36, with similar adjustments for adverse events.^16^, ^25^

Patients receiving Carbo every three weeks underwent 35 sessions of RT with intravenous Carbo (Area Under the Curve ‒ AUC 5) on days 1, 22, and 43, with dose adjustments for grade 3 or 4 adverse events.^26^ Those receiving weekly Carbo had 35 sessions with intravenous Carbo (AUC2) on days 1, 8, 15, 22, 29, and 36,^27^ with similar adjustments for adverse events.^26^ CTRT regimens (every three weeks or weekly) were determined based on individualized patient and supervisor preferences, while the decision between CDDP and Carbo was influenced by the glomerular filtration rate cutoff (> 60 mL/min/1.73 m^2^ for CDDP and < 60 mL/min/1.73 m^2^ for Carbo) and potential contraindications to the use of CDDP.^28^ RR, including Complete Response (CR), Partial Response (PR), Stable Disease (SD) and Disease Progression (DP) were defined by the Response Evaluation Criteria in Solid Tumors (RECIST) criteria.^29^ Grade 3 or 4 adverse events were documented according to the National Cancer Institute Common Terminology Criteria for Adverse Events (NCI CTCAE v5.0 2022) standards.^30^

### Survival analysis and ethical assessment

Dates of diagnosis, progression/recurrence, death, and last follow-up of patients were recorded. Event-Free Survival (EFS) and Overall Survival (OS) were determined from diagnosis to specific events (progression/recurrence/death for EFS, death for OS) or last follow-up. Exploratory analysis of clinic pathological factors and their association with survival was conducted descriptively. The *t*-test was applied for quantitative variables, while Fisher or chi-square tests were used for categorical variables in analyses amongst treatment groups. EFS and OS estimates were obtained using the Kaplan-Meier method, with hypothesis testing through log-rank test. Univariate and multivariate Cox regression analyses were conducted to identify factors associated with survival outcomes. All variables with *p-*value < 0.10 in univariate analysis were subjected to multivariate regression in stepwise approach. The RStudio (version 2023.06.1) was used for statistical estimates, and differences between groups were considered significant when the *p*-value was inferior to 0.05.

The study received approval from the Ethics Research Committee (CEP) of UNICAMP, with a waiver of the Informed Consent Form due to the retrospective nature and confidential character of the information (CAAE: 42713620.7.0000.5404; approval number: 4.579.705).

## Results

**Fig. 1:**
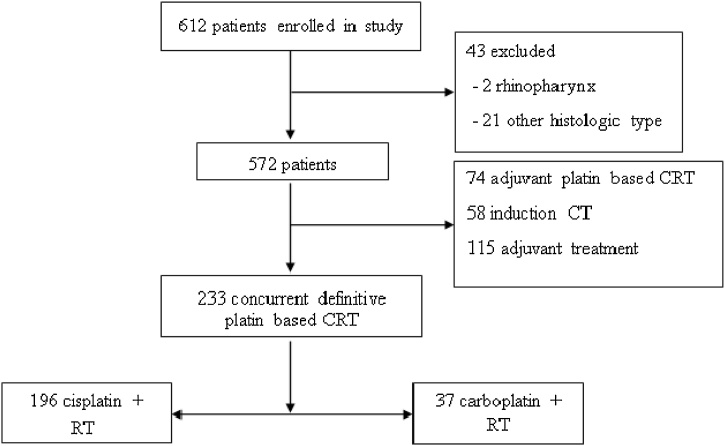
CONSORT diagram for squamous cell carcinoma of head and neck patients’ selection. RT, Radiotherapy; CRT, Chemoradiotherapy.

We identified 717 patients with HNSCC attended at our service during the study period. Among these, 475 were excluded for undergoing treatments different from the study’s aims. Nine patients treated outside the established time interval, with tumors of histological types other than SCC, with lack of medical records for data collection or with insufficient data were excluded from the study. Thus, 233 patients with locoregionally advanced HNSCC undergoing definitive treatment with platinum-based CTRT were analyzed, with 196 receiving concurrent platinum-based CTRT as definitive treatment (Fig. 1).

The median age of patients enrolled in study was 60 years. Most were males, active or former smokers and drinkers, with ECOG status 0 or 1 and no comorbidities, and presented moderately differentiated and advanced tumors. Tumors were equally distributed in oral cavity, pharynx and larynx. RT and CDDP (every three weeks in 170 cases and weekly in 25 cases) was administered to 196 patients and 37 patients received RT plus Carbo (every three weeks in 14 cases and weekly in 22 cases). Patients treated with RT and Carbo were older, while those in the RT plus CDDP group included a higher proportion of female patients. Similar smoking history, alcohol intake, ECOG PS, comorbidities, tumor location, histological grade, grade 3 or 4 toxicities, and treatment response were seen in patients treated with RT plus CDDP and RT plus Carbo (Table 1). Near 50% of the evaluated patients presented toxicities grade 3 or 4. CR or PR was observed in 75% of the cohort, indicating significant tumor control.

**Table 1:** Clinicopathological aspects of 233 patients with locoregional advanced head and neck squamous cell carcinoma undergoing radiotherapy plus cisplatin (n = 196) or carboplatin (n = 37) as definitive protocols.

Variable	Total, n (%)	RT + CDDP, n (%)	RT + Carbo, n (%)	*p-*Value
Median age (range)	60.0 (40.0‒85.0)	59.0 (40.0‒83.0)	66.8 (49.0‒85.0)	**<0.0001**
Sex				
Female	28 (12.0)	19 (9.7)	9 (24.3)	**0.02**
Male	205 (88.0)	177 (90.3)	28 (77.8)	
Smoking				0.96
Active	93 (40.0)	79 (40.3)	14 (5.4)	
Former	128 (55.0)	107 (54.6)	21 (56.8)	
Never	12 (5.0)	10 (5.1)	2 (37.8)	
Alcohol intake				
Active	50 (21.5)	44 (22.4)	6 (16.2)	0.72
Former	141 (60.5)	117 (59.7)	24 (64.9)	
Never	42 (18,0)	35 (17.9)	7 (18.9)	
ECOG				
0‒1	201 (86.3)	173 (88.3)	28 (75.7)	0.09
2‒3	29 (12.4)	21 (10.7)	8 (21.6)	
Not available	3 (1.3)	2 (1.0)	1 (2.7)	
Comorbidity				
No	31 (13.3)	28 (14.3)	3 (8.1)	0.43
1 or more	202 (86.7)	168 (85.7)	34 (91.9)	
Tumor location				
Oral cavity	72 (30.9)	58 (29.6)	14 (37.8)	0.41
Pharynx	77 (33.0)	64 (32.7)	13 (8.1)	
Larynx	84 (36.1)	74 (37.8)	10 (27.0)	
Tumor grade				
Well differentiated	6 (2.6)	3 (1.6)	3 (8.1)	0.06
Moderately differentiated	172 (73.8)	149 (76.0)	23 (62.1)	
Poorly differentiated	25 (10.7)	20 (10.2)	5 (13.5)	
Not available	10 (11.5)	2 (5.3)	8 (16.3)	
TNM stage				
II	6 (2.6)	5 (2.6)	1 (2.7)	0.16
III	56 (24.0)	43 (21.9)	13 (35.1)	
IV	171 (73.4)	148 (75.5)	23 (62.2)	
Toxicity grade 3 or 4				
No	117 (50.2)	100 (51.0)	17 (45.9)	0.69
Yes	116 (49.8)	96 (49.0)	20 (54.1)	
Treatment response				
Complete	108 (46.3)	92 (46.9)	16 (43.2)	0.93
Partial	67 (28.7)	57 (29.1)	10 (27.0)	
Stable disease	17 (7.4)	14 (7.1)	3 (8.1)	
Disease progression	27 (11.6)	24 (12.2)	3 (8.1)	
Not available	14 (6.0)	9 (4.7)	5 (13.6)	

RT, Radiotherapy; CDDP, Cisplatin; Carbo, Carboplatin; ECOG, Eastern Cooperative Oncology Group performance status; TNM, Tumor-lymph Node-Metastasis. Bold numbers represent significant values (*p* < 0.05).

Nausea and nephrotoxicity were the most common adverse events in the overall group and was more common in the RT and CDDP subgroup (18.9% vs. 2.7%, *p* = 0.01). In the RT and Carbo group, the most frequent adverse events were anemia, mucositis, and asthenia, with anemia being more prevalent in this group than in the CDDP and RT-treated group (13.5% vs. 2.0%, *p* = 0.006) (Table 2).

**Table 2:** Grade 3 or 4 adverse events in 233 patients with locoregional advanced squamous cell carcinoma treated with radiotherapy plus cisplatin (n = 196) or carboplatin (n = 37) as definitive protocols.

Adverse event	Total group, n (%)	RT + CDDP, n (%)	RT + Carbo, n (%)	*p*-Value
All adverse effects	116 (49.8)	96 (49.0)	20 (54.1)	0.69
Asthenia	13 (5.6)	9 (4.6)	4 (10.8)	0.13
Anorexia	8 (3.4)	6 (3.1)	2 (5.4)	0.61
Mucositis	22 (9.4)	17 (8.7)	5 (13.5)	0.36
Vomiting	25 (10.7)	22 (11.2)	3 (8.1)	0.77
Nausea	35 (15.0)	32 (16.3)	3 (8.1)	0.31
Diarrhea	8 (3.4)	8 (4.1)	0 (0.0)	NC
Anemia	9 (3.8)	4 (2.0)	5 (13.5)	**0.006**
Neutropenia	12 (5.1)	11 (5.6)	1 (2.70	**0.01**
Thrombocytopenia	2 (0.8)	2 (1.0)	0 (0.0)	NC
Nephrotoxicity	38 (16.3)	37 (18.9)	1 (2.7)	**0.01**
Ototoxicity	9 (3.9)	9 (4.6)	0 (0.0)	NC
Others	2 (0.8)	2 (1.0)	0 (0.0)	NC

RT, Radiotherapy; CDDP, Cisplatin; Carbo, Carboplatin; NC, Not Calculated; Others, xerostomia, hand-foot syndrome, thromboembolic event and/or endocrinological disorder. Bold numbers represent significant values (*p* < 0.05).

The median follow-up was 23.7 mo (range: 1.6 to 87.0 mo). At the end of the study (August 2022), 130 patients had died (123 from the disease and seven from unrelated causes) and 103 patients were alive (29 with disease and 74 disease-free).

**Fig. 2:**
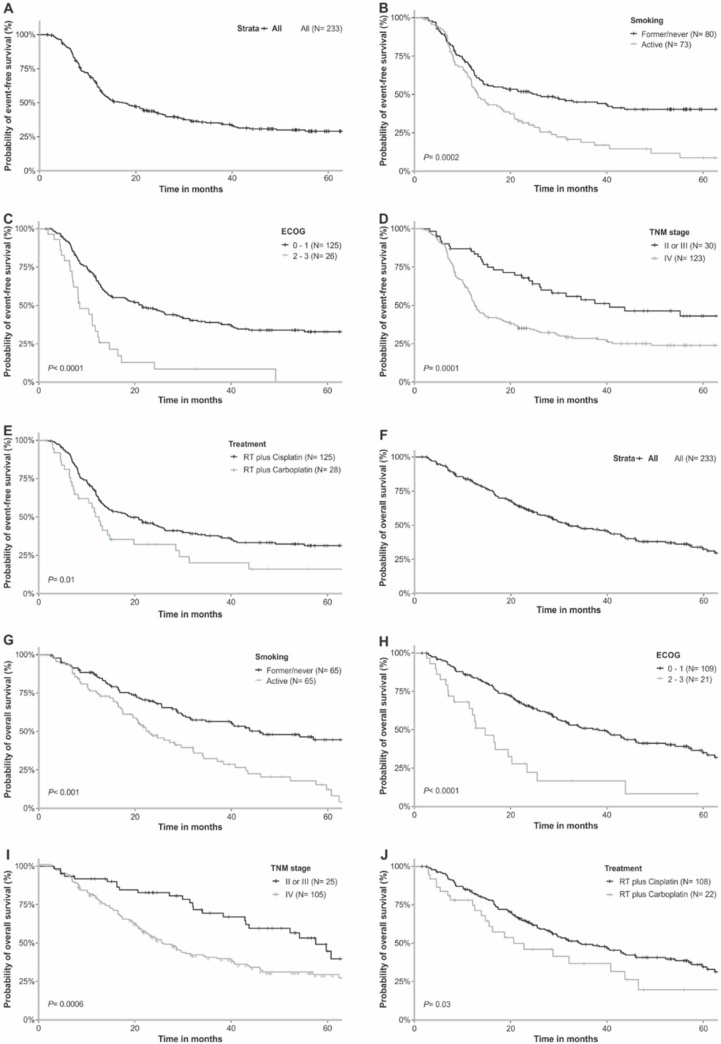
Probabilities of EFS and OS by Kaplan-Meier estimates in the total group of patients and in patients stratified by clinicopathological aspects. (A) EFS in general population. (B) EFS according to smoking habit. (C) EFS according to ECOG performance status. (D) EFS according to TNM stage. (E) EFS according to treatment. (F) OS in general population. (G) OS according to smoking habit. (H) OS according to ECOG performance status. (I) OS according to TNM stage. (J) OS according to treatment regimen. EFS, Event-free survival; OS, Overall Survival. RT, Radiotherapy. ECOG, Performance status according to Eastern Cooperative Oncology Group. TNM, Tumor-lymph Node-Metastasis.

The five-year and two-year EFS were 29.0% and 43.3%, respectively (. A). At two-year of follow-up, EFS was lower in active smokers (30.0% vs. 51.8%, *p* = 0.0002) (Fig. 2B), active drinkers (29.3% vs. 47.0%, *p* = 0.002), patients with ECOG ≥ 2 (12.9% vs. 47.7%, *p* < 0.0001) (Fig. 2C), one or more comorbidities (38.8% vs. 72.5%, *p* = 0.007), stage IV local tumor (66.0% vs. 35.1%, *p* = 0.0001) (Fig. 2D), and treatment with RT plus Carbo (32.2% vs. 45.3%, *p* = 0.01) (Fig. 2E) (Kaplan-Meier estimates). The variables remained predictors of shorter EFS in univariate Cox analysis. Multivariate Cox multivariate analysis showed that active smokers, patients with ECOG ≥ 2, stage IV local tumor, and those treated with RT plus Carbo had approximately double the risk of presenting tumor recurrence/progression or death than others, respectively (Table 3).

**Table 3:** Association of clinicopathological aspects with event-free survival and overall survival in 233 patients with head and neck squamous cell carcinoma.

Variable	Total (n)	Event-free survival					Overall survival				
		N of event	Univariate analysis		Multivariate analysis		N of event	Univariate analysis		Multivariate analysis	
			HR (95% CI)	*p*-Value	HR (95% CI)	*p-*Value		HR (95% CI)	*p-*Value	HR (95% CI)	*p-*Value
Age											
≤ 60‒years	112	79	1.21 (0.59‒1.12)	0.22	NA	NA	70	1.23 (0.57‒1.14)	0.24	NA	NA
> 60‒years	121	74	Reference				60	Reference			
Sex											
Female	28	21	1.28 (0.49‒1.23)	0.29	NA	NA	13	1.14 (0.64‒2.02)	0.65	NA	NA
Male	205	132	Reference				117	Reference			
Smoking											
Active	93	73	1.82 (1.31‒2.51)	0.0002	1.71 (1.16‒2.50)	**0.005**	65	2.17 (1.53‒3.07)	<0.0001	2.02 (1.33‒3.07)	**0.0009**
Former/never	140	80	Reference		Reference		65	Reference		Reference	
Alcohol intake											
Yes	50	41	1.79 (1.22‒2.50)	0.002	1.27 (0.83‒1.94)	0.25	39	2.04 (1.40‒2.97)	0.0002	1.37 (0.87‒2.14)	0.16
Former/never	183	112	Reference		Reference		91	Reference		Reference	
ECOG											
0‒1	201	125	Reference	<0.0001	Reference	**0.0002**	109	Reference	<0.0001	Reference	**0.0004**
2‒3	29	26	2.83 (1.84‒4.37)		2.24 (1.44‒3.47)		21	3.02 (1.87‒4.87)		2.39 (1.47‒3.88)	
Comorbidity											
No	31	13	Reference	0.007	Reference	0.13	12	Reference	0.01	Reference	0.14
1 or more	202	140	2.13 (1.20‒3.78)		1.55 (0.86‒2.79)		118	2.13 (1.17‒3.88)		1.56 (0.85‒2.88)	
Tumor location											
Oral cavity	72	45	Reference	0.93	NA	NA	35		0.54	NA	NA
Pharynx/larynx	161	108	1.02 (0.80‒1.18)				95	1.06 (0.86‒1.32)			
Tumor grade											
W + M	178	115	Reference		NA	NA	115	Reference	0.77	NA	NA
P	25	17	1.07 (0.64‒1.79)	0.77			17	1.07 (0.64‒1.79)			
TNM stage											
II + III	62	30	Reference		Reference		25	Reference		Reference	
IV	171	123	2.11 (1.41‒3.16)	0.0002	1.96 (1.29‒2.98)	**0.001**	105	2.10 (1.35‒3.25)	0.0009	2.13 (1.35‒3.37)	**0.001**
Treatment											
RT + CDDP	196	125	Reference	0.02	Reference	**0.01**	108	Reference	0.03	Reference	**0.005**
RT + Carbo	37	28	1.62 (1.07‒2.45)		1.74 (1.14‒2.68)		22	1.63 (1.03‒2.58)		1.93 (1.20‒3.10)	

HR, Hazard Ratio; CI, Confidence Interval; ECOG, Eastern Cooperative Oncology Group performance status; W, Well differentiated; M, Moderately differentiated; P, Poorly differentiated; TNM, Tumor-lymph Node-Metastasis; RT, Radiotherapy; CDDP, Cisplatin; Carbo, Carboplatin. Bold numbers represent significant values after multivariate analysis (*p* < 0.05).

The five-year and two-year OS were 32.4% and 60.6%, respectively (Fig. 2F). Active smokers (47.4% vs. 69.0%, *p* < 0.001) (Fig. 2G), active drinkers (40.4% vs. 66.0%, *p* = 0.0001), patients with ECOG ≥ 2 (22.3% vs. 65.0%, *p* < 0.0001) (Fig. 2H), one or more comorbidities (58.3% vs. 75.4%, *p* = 0.01), stage IV local tumor (82.8% vs. 52.5%, *p* = 0.0006) (Fig. 2I), and treatment with RT plus Carbo (46.1% vs. 63.0%, *p* = 0.03) (Fig. 2J) presented lower OS than others at two-year follow up (Kaplan-Meier estimates). These variables were predictors of shorter OS in univariate Cox analysis, and in multivariate Cox analysis active smokers, patients with ECOG ≥ 2, stage IV local tumor, and treatment with RT plus Carbo had approximately double the risk of evolving to death (Table 3).

At two-year of follow-up, similar OS were observed in patients stratified by age, tumor location, grade, grade 3 or 4 toxicity, type of response to treatment, and weekly or every three weeks CDDP or Carbo (Kaplan-Meier estimates).

## Discussion

In this retrospective study, toxicities, tumor control, and survival of locoregionally advanced HNSCC patients definitively treated with platin-based CTRT in a public hospital of southeast region of Brazil were evaluated.

The clinicopathological characteristics of our cohort were broadly consistent with those of patients with HNSCC reported globally,^12^, ^31^ suggesting that our sample is representative of typical cases seen in clinical practice. Nonetheless, the lack of HPV status testing in the evaluated population restricts direct comparisons regarding this key etiological factor. While tobacco and alcohol consumption are globally recognized as primary risk factors for HNSCC, alongside HPV infection,^10^, ^32^ evidence suggests that HPV-driven carcinogenesis is less prevalent in Brazilian cohorts,^33^, ^34^ consistent with findings from similar regional studies. It is essential to acknowledge this limitation early in the discussion, as it may influence the interpretation and generalizability of the study’s outcomes.

Regarding safety, toxicity of grade 3 or greater was observed in about 50% of patients treated with platinum-based CTRT. CDDP is well-documented to cause significant adverse effects, including nausea (16.3%) and nephrotoxicity (18.9%) in our study, aligning with literature reporting incidences up to 41% for nausea and 28% for nephrotoxicity.^16^, ^25^, ^26^, ^35^^,^^36^ Additionally, we observed a statistically significant difference in nephrotoxicity when compared to the group treated with RT and Carbo (18.9% vs. 2.7%, *p* = 0.01).^16^ Conversely, Carbo was associated with higher rates of anemia (13.5% vs. 2.0%; *p* = 0.006), mucositis (13.5%), and asthenia (10.8%). The use of Carbo appears to be associated with different toxicity profiles. Pignon et al. (2009), in a meta-analysis of 93 randomized trials, highlighted that the main adverse events related to Carbo included anemia and neutropenia.^19^ Lokich et al. (1998) also reported that patients treated with Carbo experienced anemia in approximately 15% of cases.^35^

The overall RR, defined as complete or partial response, was 79.9% in this cohort. There was no significant difference between the CDDP and Carbo (79.7% vs. 81.2%; *p* = 1.00). These findings align with the ConCERT trial, which reported a RR of 81% in patients receiving weekly CDDP and 78% in those receiving triweekly CDDP, showing no significant difference in RR between these schedules.^25^ Additionally, Xiang et al. (2019) observed response rates of 80% with CDDP and 78% with Carbo in their cohort, reinforcing the equivalence between these agents when combined with RT.^26^ Disease progression occurred in 11.6% of patients, with no significant difference between the RT and CDDP group and the RT and Carbo group (*p* = 0.93). This is consistent with the ConCERT trial, where disease progression rates were 12% for weekly CDDP and 13% for triweekly CDDP, again demonstrating comparable outcomes between different dosing schedules.^25^ Similarly, Xiang et al. (2019) reported progression-free survival rates of 74% for CDDP and 72% for Carbo, further supporting the similarity in efficacy between the two agents.^26^ These results highlight the robustness of both CDDP and Carbo-based CTRT regimens.

Survival outcomes in this study were comparable to those reported in similar populations. Five-year and two-year EFS rates were 29.0% and 43.3%, respectively, while OS rates were 32.4% and 60.6%. These survival probabilities are in line with previously reported findings in similar patient populations with unresectable HNSCC.^19^ For example, in the intergroup phase III trial conducted by Adelstein et al. (2003), patients treated with concurrent CRT using CDDP had a five-year OS rate of 27%, demonstrating the aggressive nature of the disease and the challenges in achieving long-term survival with standard treatments.^16^ The EFS rates, while less commonly reported in such detail, align with the trends observed in the MARCH meta-analysis, which highlighted the incremental benefits of adding CT to RT in locally advanced HNSCC.^24^

Direct comparisons between CDDP and Carbo in CTRT are yet limited. Xiang et al. (2019) compared the survival outcomes of patients treated with either CDDP or Carbo in CTRT and found similar five-year OS rates, approximately 30%, supporting the comparable effectiveness of these agents in this setting.^26^ However, in a recent meta-analysis of randomized-controlled trials, evidence suggests that CTRT with CDDP may confer greater OS compared to CTRT with Carbo, albeit accompanied by increased toxicity.^36^ However, Carbo emerges as a possible alternative for patients ineligible to CDDP, albeit associated with increased myelosuppression,^35^, ^37^ though the lack of sufficient data regarding Carbo’s radiosensitizing effect in patients with HNSCC underscores the need for further research in this area.^38^ In this present cohort, it was found that active smoking, alcohol consumption, ECOG 2 or higher, stage IV tumor, and definitive CTRT protocol with RT plus Carbo were independent predictors of worse EFS and OS. CDDP is commonly used in regimens of three cycles every three weeks, administered in conjunction with RT. The ConCERT trial also reported similar survival outcomes between weekly and triweekly CDDP regimens; further reinforcing the flexibility in treatment schedules without compromising long-term outcomes.^25^ Data from our study was underpowered to assess this question.

The findings of this study indicate that active smoking is a significant independent prognostic factor for EFS and OS. Several additional studies have consistently demonstrated that smoking negatively impacts survival outcomes. Specifically, Hilgert et al. (2009) described a significant reduction in 10-year survival rates for smokers (28.8%) compared to non-smokers (43.1%).^39^ Furthermore, Cadoni et al. (2017) elucidate that the risk of developing second primary cancer is markedly heightened in patients with advanced tumor stages, with HR of 2.75 (95% CI 1.39‒5.44). This risk is further amplified in individuals with a history of tobacco use exceeding 40 years, where the HR reaches 3.68 (95% CI 1.10‒12.30).^40^ These findings highlight the critical need to address tobacco use in treatment protocols to improve therapeutic outcomes.

In this study, alcohol consumption also emerged as an independent prognostic factor. Deleyiannis et al. (1996) demonstrated that alcohol use correlates with an increased risk of complications and adverse treatment effects.^41^ Further emphasizing this relationship, recent findings indicate that alcoholism is associated with a relative risk of 2.06 (95% CI 1.43–2.98) for increased mortality. Notably, these associations persist independently of age, primary cancer location, stage, histopathologic grade, smoking history, and the type of antineoplastic treatment, underscoring the multifaceted impact of alcohol on treatment outcomes in HNSCC patients. Addressing alcohol consumption through targeted interventions may enhance treatment efficacy and improve survival in this population.

Another critical prognostic factor identified was an ECOG of 2 or higher. Argiris et al. (2004) demonstrated that lower functional status strongly correlates with worse survival outcomes in HNSCC patients. Specifically, the hazard ratio (HR) for mortality in patients with ECOG 1 compared to ECOG 0 was 1.45 (95% CI 1.15–1.83; *p* = 0.0016), indicating a 45% higher probability of death associated with mild functional impairment.^42^ Recent evidence from Cirillo et al. (2024) highlights the prognostic significance of ECOG PS as a strong predictor of Progression-Free Survival (PFS) and OS in real-world settings. Patients with ECOG 2 had the poorest outcomes, with a median PFS of 3-mo (95% CI 3–5) and a median OS of 3-mo (95% CI 2–17), with multivariable analysis confirming association between ECOG PS and PFS and OS. In contrast, patients with ECOG 0 exhibited the most favorable outcomes, achieving a median PFS of 7-mo (95% CI 3–11) and a median OS of 14-mo (95% CI 8–not estimable). Patients with ECOG 1 showed intermediate results, with a median PFS of 5-mo (95% CI 3–not estimable) and a median OS of 7-mo (95% CI 5–not estimable).^43^ These findings emphasize the importance of incorporating functional status into therapeutic decision-making, as maintaining good performance scores can enhance treatment tolerance and improve prognosis.

This study also revealed that patients with stage IV tumors faced significantly poorer survival outcomes, as validated by previously published studies. Leoncini et al. (2018) reported that stage IV disease is associated with a markedly higher risk of mortality. Combined HNSCC cases showed an HR of 4.16 (95% CI 2.49–6.96), indicating a nearly fourfold increase in risk compared to earlier stages.^44^ Advanced-stage tumors complicate treatment, requiring more aggressive regimens that may exacerbate toxicities and compromise outcomes. The presence of metastatic disease complicates treatment approaches, necessitating more aggressive and intensive therapeutic regimens, which can further exacerbate treatment-related complications. This complexity not only impacts treatment efficacy but also contributes to a general decline in prognosis, emphasizing the urgent need for early detection and personalized treatment strategies to mitigate the adverse effects associated with advanced-stage HNSCC.

In addition, the integration of biomarker analysis into the management of HNSCC represents a promising avenue for improving patient outcomes. A recent publication highlights the immunohistochemical expression of Orphanin for predicting laryngeal SCC recurrence. Thus, although not included in this study, biomarker evaluations into clinical practice seems to enhance risk stratification, guiding treatment decisions and follow-up strategies to optimize long-term outcomes for patients with HNSCC45.^45^

It is worth highlighting that Brazil is a country of great economic contrasts,^46^, ^47^ with numerous hospitals similar to those found in developed countries and others that struggle with a lack of financial resources, particularly public hospitals, where the present study was conducted. In Brazil, the Unified Health System (SUS – *Sistema Único de Saúde*) serves more than 70% of the population; however, disparities in access to advanced treatments are substantial. While some hospitals offer cutting-edge technology, others face shortages of basic resources, contributing to late diagnoses and compromised treatment outcomes.^48^, ^49^

In developed countries, survival rates for HNSCC can exceed 60% over five years.^50^ However, in Brazil, especially in public institutions, the scenario is characterized by challenges such as delays in the initiation of treatment, with an average that can exceed 60 days between diagnosis and the first therapeutic procedure. Moreover, the completion of treatment within the recommended timelines varies significantly across regions, with concerning low rates in certain areas^51^ which contributes to an increase in complications and the necessity for more aggressive interventions. These delays and the difficulty in accessing specialized care not only compromise patient prognosis but also overload the public health system, which deals with high demand and resource scarcity.

The present study was conducted in a public hospital, where the challenges of limited financial and technological resources, coupled with high service demand, were clearly evident. These issues reflect the broader struggles faced by Brazil’s SUS. A national survey highlighted that nearly 60% of oncology patients in public hospitals experience delays in starting treatment, significantly affecting survival outcomes. Addressing these systemic inequalities is crucial to improving oncological care in Brazil, ensuring equitable treatment access, and reducing the mortality burden of head and neck cancers.

It is important to acknowledge the inherent limitations of this study, including its retrospective design, the non-randomized allocation of patients to treatment arms, and the small number of patients treated with RT and Carbo. These factors may have limited the statistical power to detect significant differences between groups.

## Conclusion

The identification of these independent predictors highlights the complexities of managing HNSCC, emphasizing the necessity of a comprehensive approach to treatment planning and patient support. Addressing modifiable factors such as smoking and alcohol consumption, along with careful consideration of performance status and disease stage, can lead to better survival outcomes for patients in this challenging clinical setting. CDDP is commonly used in regimens of three cycles every three weeks, administered in conjunction with RT. However, Carbo emerges as an alternative for patient’s ineligible to CDDP, albeit associated with increased myelosuppression. Our data indicate RT and CDDP as a good option for definitive treatment of patients with locoregionally advanced HNSCC in public hospitals, but a prospective and randomized phase 3 study is required to establish the ideal treatment for those patients. Furthermore, these findings have the potential to shape public health policies, promoting improved access to effective treatments and addressing disparities in oncological care.

## Justification for the number of authors

The number of authors exceeds the journal’s limit of seven; however, we request the inclusion of all 11 authors due to their significant contributions. This study involved a multidisciplinary team of oncologists, radiotherapists, and surgeons, each playing a critical role in the development of the article. Patients in our study were attended to and selected by Sousa HSV, Horita VN, Novaes DML, Perin MY, Macedo LT, and Lima CSP in the Clinical Oncology Service; by Teixeira DNA and Chone CT in the Department of Ophthalmology and Otolaryngology; and by Gruenwaldt J and Pereira EB in the Radiotherapy Service. Lourenço GJ performed the statistical analyses. All authors have reviewed and approved the manuscript.

## Funding

The authors received no specific funding for this work.

## Declaration of competing interest

The authors declare no conflicts of interest.
